# Additional improvement in regional myocardial ischemia after intracardiac injection of bone marrow cells during CABG surgery

**DOI:** 10.3389/fcvm.2023.1040188

**Published:** 2023-02-07

**Authors:** Luís Henrique Wolff Gowdak, Isolmar Tadeu Schettert, Carlos Eduardo Rochitte, Leonardo P. de Carvalho, Marcelo Luiz Campos Vieira, Luís Alberto Oliveira Dallan, Sérgio Almeida de Oliveira, Luiz Antonio Machado César, José Oscar Reis Brito, Luiz César Guarita-Souza, Antonio Carlos Campos de Carvalho, Jose Eduardo Krieger

**Affiliations:** ^1^Laboratory of Genetics and Molecular Cardiology, Heart Institute (InCor-HCFMUSP), University of São Paulo Medical School, São Paulo, Brazil; ^2^Department of Cardiovascular Surgery, National Institute of Cardiology, Rio de Janeiro, Brazil; ^3^Department of Cardiovascular Surgery, Pontifical Catholic University of Paraná, Curitiba, Brazil; ^4^Cell Technology Center, National Institute of Cardiology, Rio de Janeiro, Brazil; ^5^Carlos Chagas Filho Biophysics Institute, Federal University of Rio de Janeiro, Rio de Janeiro, Brazil

**Keywords:** myocardial ischemia, coronary artery disease, bone marrow cells, coronary artery bypass graft surgery (CABG), cardiovascular magnetic resonance, stress induced myocardial ischemia

## Abstract

**Background:**

Post-procedure residual ischemia is associated with worse prognosis in patients with coronary artery diasease (CAD).

**Objective:**

We evaluated whether autologous bone marrow-derived cells (BMC) contribute to additional reduction in regional stress-induced myocardial ischemia (SIMI) in patients undergoing incomplete coronary artery bypass graft surgery (CABG).

**Methods:**

In a double-blind, randomized, placebo-controlled trial, we enrolled 143 patients (82% men, 58 ± 11 years) with stable CAD and not candidates for complete CABG. They received 100 million BMC (*n* = 77) or placebo (*n* = 66) injected into ischemic non-revascularized segments during CABG. The primary outcome was improvement on SIMI quantified as the area at risk in injected segments assessed by cardiovascular magnetic resonance (CMR) 1, 6, and 12 months after CABG.

**Results:**

The reduction in global SIMI after CABG was comparable (*p* = 0.491) in both groups indicating sustained beneficial effects of the surgical procedure over 12 month period. In contrast, we observed additional improvement in regional SIMI in BMC treated group (*p* = 0.047). Baseline regional SIMI values were comparable [18.5 (16.2–21.0) vs. 18.5 (16.5–20.7)] and reached the lowest values at 1 month [9.74 (8.25; 11.49) vs. 12.69 (10.84; 14.85)] for BMC and placebo groups, respectively. The ischemia’s improvement from baseline represented a 50% difference in regional SIMI in favor of the BMC transplanted group at 30 days. We found no differences in clinical and LVEF% between groups during the 12 month follow-up period. The 1 month rate of major adverse cerebral and cardiovascular events (MACCE) (*p* = 0.34) and all-cause mortality (*p* = 0.08) did not differ between groups 1 month post intervention.

**Conclusion:**

We provided evidence that BMC leads to additional reduction in regional SIMI in chronic ischemic patients when injected in segments not subjected to direct surgical revascularization. This adjuvant therapy deserves further assessment in patients with advanced CAD especially in those with microcirculation dysfunction.

**Clinical trial registration:**

https://clinicaltrials.gov/, identifier NCT01727063

## 1. Introduction

Coronary artery bypass graft surgery (CABG) remains the most performed procedure in cardiovascular surgery (1). The importance of achieving complete revascularization and its impact on the long-term prognosis of patients with multivessel coronary artery disease (CAD) has been addressed in many observational studies and subgroup analyses of randomized clinical trials. In a meta analysis comprising 35 studies and almost 90,000 patients, complete revascularization was associated with lower long-term mortality risk, myocardial infarction and repeat coronary revascularization (2). Based on myocardial perfusion serial testing after PCI for stable angina in the ACME trial (3), normalization of perfusion abnormalities was associated with improved survival compared to patients with persistent ischemia. In the COURAGE trial (4), the extent of residual ischemic myocardium increased the risk of death or myocardial infarction (MI) at 5 years. Nevertheless, the rates of incomplete revascularization varied from 16% in the ARTS I trial (5) to 36% in the MASS II trial (6) or 43% in the SYNTAX CABG trial (7), indicating an yet unmet clinical need.

In myocardial ischemia animal models, stem cell-induced neoangiovasculogenesis and decreased inflammation are associated with increased myocardial perfusion. This response is due to a not fully characterized paracrine effect influencing the development of new vessels and endothelial dysfunction improvements (8–11). Nevertheless, the rate of cell homing is poor and influenced by several factors including the route of delivery and number of cells injected, which has not been thoroughly investigated (12–14), thereby affecting cell-based strategies. Moreover, new vessels presumably induced by cell therapy should remain functionally active, and not provide a transient response associated with an incomplete vessel formation, to favorably impact chronic ischemic cardiomyopathies (15).

Most of the initial studies using autologous bone marrow mononuclear cells (BMC) during CABG focused on cell therapy effects on left ventricular function recovery, which provided modest benefit (16–18). Conversely, few studies addressed the increase in myocardial perfusion and endothelial dysfunction improvements after injection of BMC in patients with severe CAD undergoing CABG. We and others have demonstrated that autologous BMC injection into ischemic, viable myocardium in a small series of patients undergoing incomplete CABG is safe (19) and leads to an increase in myocardial perfusion regardless of number of successfully implanted grafts (20). Thus, in the present MiHeart/IHD trial we hypothesized that intramyocardial injection of autologous BMC, adjuvant to CABG, provides additional regional reduction in SIMI.

## 2. Materials and methods

### 2.1. Study overview

MiHeart/IHD study was a multicenter, randomized, double-blind, placebo-controlled trial performed at eight different Brazilian sites (21). Patients were randomly assigned 1:1 *via* computer-generated block randomization using R software, version 1.9.0 (R Foundation for Statistical Computing),^1^ with variable block size (blocks of 2, 4, or 6 patients), to receive either autologous BMC or placebo (saline solution) during surgery. Randomization took place after bone marrow aspiration with the patient already in the operating room. All investigators related to patient‘s care or myocardial perfusion study analysis were blind to the allocated groups of randomization. The only unblinded investigator was the Hematologist, whose sole task in the trial was to support the preparation of the injected material.

The study protocol followed the recommendations of the Helsinki Declaration and Good Clinical Practice norms on medical research in humans. The protocol was approved by National Committee of Ethics in Research and local Institutional Review Board from all participating centers. All patients provided a signed, written, informed consent. The trial was sponsored by the Brazilian Ministry of Health (Process #0-1-04-0967-0-0) and registered at clinicaltrials.gov (NCT01727063).

### 2.2. Inclusion and exclusion criteria

Patients were eligible if the following criteria were met: (1) age between 18 and 80 years; (2) symptoms of angina or angina equivalent due to obstructive CAD documented by invasive angiography; (3) demonstrable myocardial ischemia by two different imaging methods; (4) unsuitability for complete myocardial revascularization according to the Heart Team; patients could be eligible if anticipated that myocardial perfusion might not be adequately restored due to poor distal beds.

Patients were excluded if any of the following were present: (1) LVEF < 25% assessed by echocardiogram; (2) life expectancy below 1 year; (3) cancer in the past 5 years or any blood disorders; (4) severe heart disease of other etiologies; (5) ACS in the past 3 months before surgery, or (6) chronic kidney disease stage five requiring dialysis.

### 2.3. Bone marrow cell preparation and isolation

All patients, regardless of the assigned group, underwent bone marrow aspiration. Immediately before surgery, 100 mL of bone marrow aspirate was obtained from the right posterior iliac crest and heparinized. The cell suspension was isolated by density gradient centrifugation on Ficoll-Paque Plus™ (GE Healthcare, Pittsburgh, PA, USA) and washed with heparinized saline. After cell counting using the Türk’s solution, a minimum of 100 million cells were resuspended in 4 mL normal saline, placed in four 1-mL syringes ready for injection. Cell viability assessed by trypan blue exclusion assay needed to be greater than 90% to be deemed adequate for injection. The different BMC sub-populations were assessed in all samples using a standard panel of monoclonal antibodies including VEGFR2 (KDR/Flk-1), CD34, CD117, CD3 antibody, CD4, CD8, CD15, CD19, CD45, CD56, and Stro-1. The percentage of cells expressing CD34 + marker of human hematopoietic stem cells was 1.6 ± 0.8 in BMC-treated patients.

### 2.4. Intramyocardial autologous BMC cell injection during CABG

Coronary artery bypass graft surgery was performed during cardiopulmonary bypass and warm blood cardioplegic arrest. Once all bypasses had been completed, 20 aliquots (0.2 mL) of BMC suspension or placebo were injected into non-revascularizable ischemic myocardium supplied by a main coronary artery. The 1 ml syringes used for injection were carefully prepared to prevent the surgical team from identifying the injected solution in both groups. The Cardiovascular Surgeon prospectively decided which segments ought to be injected after careful coronary angiography analysis before randomization. The injections were performed in the cardioplegic heart and special care was taken to prevent backflow after cell delivery by gentlly pressuring the injection site for few seconds.

### 2.5. Primary endpoint

The primary outcome was improvement in regional stress-induced myocardial ischemia (SIMI) quantified as% of the area at risk in injected segments assessed by cardiovascular magnetic resonance (CMR) 1, 6, and 12 months after CABG. Among the non-invasive stress methods, SIMI by CMR is recognized as an accurate technique to detect inducible myocardial ischemia and infarction with high sensitivity and specificity (22). Moreover, several large studies have shown its prognostic value for predicting CV events (23).

We hypothesized that BMC-treated segments have a higher regional Δ reduction% (Myo _ischemia at any given time point_ – Myo _ischemia at baseline_) compared to the placebo. Secondary endpoints included global Δ reduction%, improvement in LV function, and a decrease in angina functional class. The primary safety endpoint was major cerebral and cardiovascular adverse events (MACCE) during the first month after surgery.

### 2.6. Stress induced myocardial ischemia (SIMI) by cardiovascular magnetic resonance imaging (CMR)

The CMR exam was performed in 114 patients (BMC group = 59 pts and placebo group = 55 pts) following a standard protocol that included LV short and long-axis cine images acquisition (steady-state free precession – SSFP sequence) and late gadolinium enhancement. First-pass myocardial perfusion was acquired in the LV short-axis plane and obtained 2–3 min after pharmacological stress using dipyridamole at 0.56 mg.kg^–1^ injected over 4 min. A single dose of 0.05 mM.kg^–1^ of non-ionic, low-osmolar Gd-based contrast agent was injected into the antecubital vein by power injector at a rate of 5 mL.s^–1^ followed by 20 mL saline flush. Aminophylline was intravenously injected immediately after the stress perfusion image sequence. The heart was divided into 17 segments for myocardium perfusion assessment (20), which was determined not only in injected ischemic segment but also in non-injected myocardial segments; each segment was scored as presenting normal perfusion (0) mild (1), moderate (2) or severe (3) perfusion defect. The % of global ischemic burden (Myo _ischemia%_) was calculated as follows:


GlobalMyo%ischemia=(ΣMyo/ischemiascore51)×100

Where 51 represents the maximal global ischemic score (17 segments × 3 = 51). The % of regional ischemic burden (Myo _ischemia%_) in injected segments was calculated as follows:


RegionalMyo%ischemia=(ΣMyo/ischemia⁢scoren×3)×100

Where n = number of injected segments. All myocardial perfusion studies by CMR were analyzed blindly by a core lab after collecting scans. Investigators examining the images were not aware of which intervention group or time point the images belonged. Moreover, they were also unaware of which myocardial segments had been injected or revascularized.

### 2.7. Statistical analysis

Data analyses were performed using a commercially available statistical package (IBM Corp., Released 2011. IBM SPSS Statistics for Windows, Version 20.0. Armonk, NY: IBM Corp). The results for continuous variables are presented as mean ± SD, and for categorical variables as percentages. All datasets were tested for normality using the Shapiro–Wilk test. Global and regional SIMI comparisons were analyzed using generalized linear mixed models. Interaction between group and time was included in the analysis and significance was considered for a *P* ≤ 0.01 (24). Categorical data were compared using Fisher’s exact test. In patients undergoing complete myocardial revascularization, because myocardial segments previously selected as targets were injected with either BMC or placebo, an intention-to-treat analysis was performed.

## 3. Results

### 3.1. Enrollment and characteristics of the patients

**FIGURE 1:**
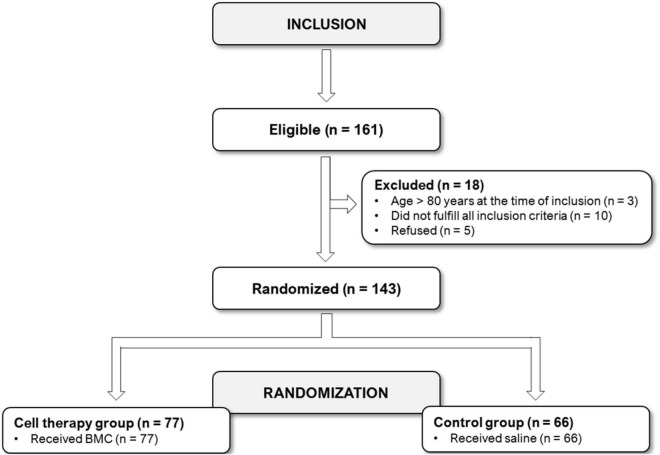
Patient’s flowchart.

We assessed for eligibility 161 patients, and 143 were randomized, 77 assigned to BMC and 66 to placebo (Figure 1). The two groups were well matched at baseline (Table 1). Patients were predominantly middle-aged men on optimal medical therapy for symptom control and cardioprotection. A significant proportion of patients had a previous myocardial revascularization procedure, and LV function was preserved or slightly compromised and did not change after CABG (Supplementary Table 1). Incomplete myocardial revascularization was performed in 90 patients (63%), 49 in BMC group (64%) and 41 (62%) in placebo group (*P* = 0.86). The remaining 53 (37%) patients underwent complete revascularization despite poor distal arterial beds and followed the intention to treat study design, meaning that target myocardial segments were injected as planned with BMC or placebo, regardless the vascular graft placement.

**TABLE 1:** Baseline clinical features.

Variable	BMC group (*N* = 77)	Placebo group (*N* = 66)	*P*-value
Age (years-old)	59 ± 11	57 ± 11	0.17
Male sex (*N*, %)	67 (87)	51 (77)	0.18
BMI (kg.m^–2^)	28.4 ± 4.3	27.2 ± 3.8	0.11
Heart rate (bpm)	71 ± 11	70 ± 11	0.58
SAP (mmHg)	129 ± 20	132 ± 19	0.42
DAP (mmHg)	80 ± 13	79 ± 14	0.67
Hypertension (*N*, %)	62 (80)	46 (70)	0.34
Diabetes (*N*, %)	32 (42)	21 (32)	0.38
Dyslipidemia (*N*, %)	48 (62)	48 (72)	0.37
Smoking (*N*, %)	32 (42)	31 (47)	0.53
Previous PCI (*N*, %)	22 (29)	20 (30)	0.88
Previous CABG (*N*, %)	29 (38)	29 (44)	0.80
LVEF (%)	52 ± 12	47 ± 12	0.052
CCS class	2.2 ± 0.8	2.3 ± 0.9	0.95
NYHA class	1.4 ± 0.7	1.5 ± 0.8	0.57
**Medications (*N*, %)**			
Antiplatelets	76 (99)	65 (99)	0.91
Statins	75 (97)	64 (97)	0.88
β-blockers	62 (80)	56 (85)	0.50
Calcium channel blockers	18 (23)	11 (17)	0.32
ACE inhibitors	55 (71)	49 (74)	0.71
Nitrates	20 (26)	18 (27)	0.86
Antidiabetics	30 (39)	20 (30)	0.28

BMC, bone marrow cells; BMI, body mass index; SAP, systolic arterial pressure; DAP, diastolic arterial pressure; PCI, percutaneous coronary intervention; CABG, coronary artery bypass graft surgery; LVEF, left ventricular ejection fraction; CCS, canadian cardiovascular society; NYHA, new york heart association.

### 3.2. BMCs transplantation

We found no differences in BMCs sub-populations among the placebo or treated group using a standard panel of monoclonal antibodies against VEGFR2 (KDR/Flk-1), CD34, CD117, CD3 antibody, CD4, CD8, CD15, CD19, CD45, CD56, and Stro-1. The percentage of cells expressing CD34 + marker of human hematopoietic stem cells, believed to contribute to neoangiovasculogenesis, averaged 1.6 ± 0.8% in the samples from BMC-treated patients.

### 3.3. Stress induced myocardial ischemia (SIMI) assessment by cardiovascular magnetic resonance imaging (CMR)

**FIGURE 2:**
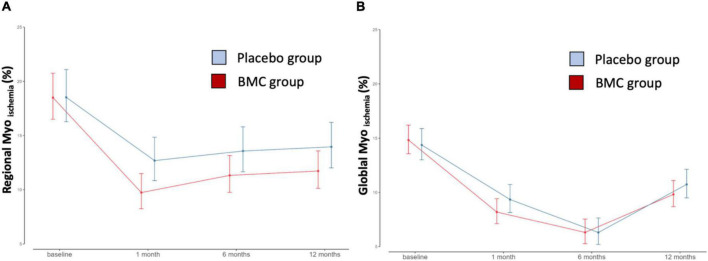
Regional and global stress-induced myocardial ischemia (SIMI). **(A)** Regional stress-induced ischemia (*P*-value = 0.047); **(B)** global stress-induced ischemia in different time points after CABG in patients receiving BMC vs. placebo group (*P*-value = 0.491).

The significant reduction in global SIMI after CABG was comparable (*p* = 0.491) in both groups indicating sustained beneficial effects of the surgical procedure over the 12 month period (Figure 2 and Table 2). In contrast, we observed additional improvement in regional SIMI only in BMC treated group (*p* = 0.047) (Figure 2 and Table 2). Baseline regional SIMI values were comparable [18.5 (16.2–21.0) vs. 18.5 (16.5–20.7)] and reached the lowest values at 1 month [9.74 (8.25; 11.49) vs. 12.69 (10.84; 14.85)] for BMC and placebo groups, respectively (Table 2). These results show a 50% improvement in reducing ischemia as indicated by the difference in regional SIMI in favor of the BMC transplanted group at 30 days.

**TABLE 2:** Effect of BMC on regional and global stress induced myocardial ischemia (SIMI).

(A)						
	Group	Baseline	1 month	6 months	12 months	*P*-value
Regional Myo _ischemia%_	BMC	18.50 (16.50; 20.76)	9.74 (8.25; 11.49)	11.33 (9.76; 13.16)	11.73 (10.12; 13.59)	0.047
	Placebo	18.53 (16.27; 21.09)	12.69 (10.84; 14.85)	13.58 (11.66; 15.81)	13.96 (12.02; 16.22)	
Global Myo _ischemia%_	BMC	14.83 (13.57; 16.20)	8.19 (7.12; 9.42)	6.31 (5.28; 7.55)	9.82 (8.69; 11.10)	0.491
	Placebo	14.38 (13.01; 15.88)	9.35 (8.14; 10.73)	6.31 (5.20; 7.65)	10.74 (9.51; 12.14)	
**(B)**						
	**Group**	1 month – baseline*		6 months – baseline*		12 months – baseline*
Regional Myo _ischemia%_	BMC	−8.76 (−11.46; −6.07)		−7.17 (−9.79; −4.56)		−6.77 (−9.38; −4.16)
	Placebo	−5.84 (−8.82; −2.86)		−4.95 (−7.90; −2.00)		−4.56 (−7.47; −1.66)
Global Myo _ischemia%_	BMC	−6.63 (−8.63; −4.64)		−8.51 (−10.53; −6.49)		−5.01 (−7.00; −3.02)
	Placebo	−5.03 (−7.17; −2.89)		−8.07 (−10.26; −5.88)		−3.63 (−5.76; −1.51)

(A) Global and regional SIMI values expressed as average and 95% confidence intervals and between group *p*-value.

(B) Global and regional SIMI differences based on the difference between the time point and baseline values expressed as average changes and 95% confidence intervals.

*All *P*-values < 0.001.

### 3.4. Angina CCS, NYHA classification, and LVEF (%) after CABG

We observed no significant differences between the groups during the 12 month follow-up period nor in clinical or echocardiographic features including angina, chronic coronary symptoms, NYHA classification, or LVEF% (Supplementary Table 1).

### 3.5. Safety

The rate of adverse events at 12 months after CABG did not differ between the treated versus the placebo group, especially MACCE (*p* = 0.34) and all-cause mortality (*p* = 0.08) that also remained the same at 1 month post-cell transplantation in both groups (Supplementary Table 2).

## 4. Discussion

Bone marrow-derived cells have been administered in humans by different routes for decades and highterto have not raised concerns about safety issues. In contrast, efficacy, measured as improvement of cardiac left ventricle ejection fraction, has shown only modest and non-sustained improvements (16–18). In the present study, we provided evidence that BMCs treatment improves stress-induced myocardial ischemia using CMR, which is considered one of the most reliable non-invasive methods to evaluate microvascular ischemia. These results are consistent with the notion that BMSc stimulate local neoangiovasculogenesis and/or improve coronary microvascular dysfunction. These findings are further supported by data obtained in a subgroup analyses of the present study where we observed that BMCs transplantation improves coronary flow reserve in ischemic non-revascularized myocardium (25).

A major challenge in the area is that neither dosage or injection routes have been standardized as usual for drug development. Pre-clinical evidence indicates that retention associated to BMCs direct injection in the myocardium is low (less than 10%), and very low or negligible when delivered by other routes (13, 14). Other aspect to consider is related to the choice of surrogate endpoints, such as left ventricle ejection fraction improvement, that has been selected early on when it was believed that adult multipotent stem cells could give rise to cardiomyocytes and regeneration. This concept was not confirmed in later studies, but still the majority of the meta analyses focuses on the improvement of cardiac function as measured by left ventricle ejection fraction (16–18). In this context, most of the clinical trials were not designed to assess the more likely effects of cell transplantation on neoangiovasculogenesis or improvements in microcirculation dysfunction consistent with recent evidence pointing to a mechanism of action related to paracrine effects associated with adult multipotent stem cells transplantation (9, 26).

The REPAIR-AMI long-term follow-up at 2 and 5 years, one of the most long-lasting follow-up analysis after BMC injection, demonstrates favorable clinical outcomes, including cardiovascular death and rehospitalization for heart failure (27). The Doppler substudy of the REPAIR-AMI trial suggested cell injection-induced improvement in coronary flow reserve, which was blunted after AMI, whereas in the placebo group there was significant less improvement (28). At 4-month follow-up, CFR in infarct artery slightly improved in placebo group, but was markedly increased by 90% in BMC-treated patients. Likewise in the present study, there was improvement in the minimal microvascular resistance during maximal hyperemia associated with BMC therapy. It is tempting to speculate that that functional improvement in microvascular dysfunction at early stage after BMC transplantation may lead to long-term benefits. We also observed a reduction in SIMI accompanied by improvement in CFR associated with BMC transplantation (25), indicating that the recovery and/or improvement in the microcirculation is a relevant end-point to be targeted.

Microvascular dysfunction accompanies the majority of pathologies of failing organs and tissues. Coronary microvascular dysfunction encompasses several pathogenetic mechanisms involving coronary microcirculation and plays a major role in determining myocardial ischemia and heart failure development (29, 30). BMCs may modulate the microvascular endothelial and metabolic functions, which may contribute to ameliorate myocardial tissue perfusion, resulting in the recovery of the coronary microvascular function, as evidenced by an improvement in coronary flow reserve (31). In this study, we focused on assessing the effects of BMCs on tissue perfusion and its impact on the ischemic burden of the ischemic non-revascularized cardiac segments, especially considering that the study group comprised mild-low risk patients with preserved LVEF. The data demonstrated that BMC injection provided a significant improvement in decreasing regional SIMI compared to placebo over the 12 month period. Moreover, the greatest improvement in the regional ischemia was observed 30 days post-transplatation, representing a 50% improvement compared to the placebo group that benefited only from the CBGE suggesting that more efforts may be directed at increasing cell retention and/or strategies to augment the frequency of the cell delivery.

Interestingly, the results of the COURAGE and BARI-2D trials randomized patients with stable ischemic heart disease after optimal medical therapy (OMT) to a routine revascularization failed to demonstrate MACCE reduction. One may speculate that despite improvement in ischemic burden, large vessels revascularization has no added impact on hard outcomes, which implies the existence of additional factors affecting the outcomes. It may be counterintuitive that routine revascularization for stable ischemic heart disease has no impact on death or MI rates, even though the intervention “fixes” or bypasses the stenosis and relieves ischemia. Nevertheless, it is important to emphasize that a relatively high proportion of participants in the COURAGE and BARI-2D trials remained with moderate to severe residual ischemia after revascularization. These findings indicate a potential benefit of adding strategies targeting neoangiovasculogenesis and/or microvascular dysfunction to complement epicardial vessel revascularization therapy. While the present study did not disclose the underlying patho-physiological mechanisms related to BMCs-induced SIMI reduction, there is a well-established inverse association between coronary flow reserve and atherosclerotic disease progression (32).

Mounting evidence suggests that adult multipotent stem cells may exert paracrine effects by secreting cardio-protective factors (9, 26). These secreted factors may stimulate vascular growth and remodeling, attenuate fibrosis, modulate inflammation, regulate cell differentiation and survival, and recruit resident stem or progenitor cells. Recently, studies have shown that these factors may be clustered into extracellular membrane vesicles, including exosomes and microsomes, which can then transfer proteins, lipids, RNA, and microRNAs to mediate cardioprotection (33, 34). One may speculate that these mechanisms contributed to our findings, but it will be critical to assess the SIMI reduction impact on MACCE in long-term follow-up, which remains to be done.

Altogether, we provided evidence for a BMC-induced improvement in SIMI in ischemic non-revascularized cardiac segments, implying neoangiovasculogenesis and/or restoration of microvasculature function to explain the ischemia reduction. Considering that these results were obtained with a single intramyocardial BMC injection, it will be critical to focus on approaches to increase cell retention and extend the actions of the beneficial effects as an adjunct therapy for ischemic cardiac areas to complement epicardial flow restoration.

### 4.1. Study limitations

The regional analysis was performed by matching the myocardial segmentation map used for perfusion studies with surgeon’s description of which segments had been injected. We acknowledge that some imprecisions may have occurred in pairing the surgeons’ reports with that map. Despite our efforts, there was a decrease in the number of patients that completed the MRI series during the study. This is somewhat expected since claustrophobia and anxiety related symptoms are frequent and known causes of failure in completing postoperative MRI (35). Nevertheless, we provided compelling and robust results despite the losses of procedures in some patients. The adverse events during the 12 month observational period and MACCE at the first month post-cell transplantation remained the same in the experimental and placebo groups. Despite this finding, a major challenge for the groups, scientific societies and regulatory boards is the development of guidelines to standardize these cell procedures as to minimize the possibility of side effects and to enable the comparison of the reported results. Finally, a longer follow-up with an appropriate number of patients would be necessary to determine the combined strategy’s impact on clinically relevant endpoints such as mortality, non-fatal myocardial infarction or rehospitalizations.

## 5. Conclusion

We provided evidence that intramyocardial injection of BMC reduces SIMI in ischemic non-revascularized cardiac segments suggesting that this strategy may target the affected microcirculation by activating cardiac neoangiovasculogenesis and/or improving endothelial dysfunction. These findings suggest that adult multipotent stem cells transplantation has a potential as an adjuvant therapy to complement flow restoration of ischemic myocardium.

## Data availability statement

The original contributions presented in this study are included in this article/Supplementary material, further inquiries can be directed to the corresponding author.

## Ethics statement

The studies involving human participants were reviewed and approved by the Brazilian National Committee of Ethics in Research and local Institutional Review Boards from all participant centers. The patients/participants provided their written informed consent to participate in this study.

## Author contributions

LG, SO, and JK designed and conceptualized the study. LG, MV, LD, SO, LC, JB, and LG-S selected and assisted the patients. LG, IS, and CR collected and analyzed the data. LG, CR, and JK prepared the first draft. LPC and JK prepared the final version. AC, SO, and JK obtained the funds and coordinated the study. All authors contributed to the article and approved the submitted version.

## Footnotes

1
http://www.r-project.org
