# Supplementary material for: Additional improvement in regional myocardial ischemia after intracardiac injection of bone marrow cells during CABG surgery
Source: Front Cardiovasc Med. 2023 Feb 7;10:1040188. doi: 10.3389/fcvm.2023.1040188 (PMC9941147; doi:10.3389/fcvm.2023.1040188)
Supplement: Supplementary file 1 [file Data_Sheet_1.PDF]

Suppl. Table 1. Clinical status and LVEF 12 months after CABG.

**Variable**	**BMC group (N=77)**	**Placebo group (N=66)**	**P-value**
**Canadian Cardiovascular Society**			
Baseline	2.2±0.8	2.3±0.9	0.95
1 month	1.3±0.5	1.3±0.5	0.97
6 months	1.2±0.4	1.1±0.3	0.19
12 months	1.2±0.5	1.1±0.4	0.24
**New York Heart Association**			
Baseline	1.4±0.7	1.5±0.8	0.57
1 month	1.1±0.3	1.1±0.3	0.28
6 months	1.1±0.3	1.0±0.2	0.19
12 months	1.1±0.4	1.1±0.3	0.46
**LVEF (%)**			
Baseline	52±12	47±12	0.052
1 month	54±10	50±14	0.12
6 months	53±9	51±11	0.64
12 months	55±11	52±11	0.17

Suppl. Table 2. Adverse events at 12 months after CABG.

Adverse event	BMC group (N=77)	Placebo group (N=66)	P-value
Pulmonary infection (n, %)	1 (1.3%)	5 (7.6%)	0.06
Septic shock (n, %)	4 (5.2%)	0 (0.0%)	0.06
Surgical wound infection (n, %)	2 (2.6%)	0 (0.0%)	0.19
Pulmonary embolism (n, %)	1 (1.3%)	0 (0.0%)	0.35
Systemic inflammatory response syndrome (n, %)	1 (1.3%)	0 (0.0%)	0.35
Acute sinusitis (n, %)	1 (1.3%)	0 (0.0%)	0.35
Bloodstream infection (n, %)	1 (1.3%)	0 (0.0%)	0.35
Pulmonary embolism (n, %)	1 (1.3%)	0 (0.0%)	0.35
Cardiogenic shock (n, %)	1 (1.3%)	1 (1.5%)	0.91
All-cause mortality (n, %)	6 (7.8%)	1 (1.5%)	0.08
Stroke (n, %)	2 (2.6%)	0 (0.0%)	0.19
Acute myocardial infarction (n, %)	0 (0.0%)	1 (1.5%)	0.28
Cardiovascular mortality (n, %)	3 (3.8%)	1 (1.5%)	0.39
MACCE	5 (6.4%)	2 (3.0%)	0.34
All-cause mortality (n, %)	6 (7.8%)	1 (1.5%)	0.08

Major adverse cerebral and cardiovascular events (MACCE).
